# 3,7-Dihydroxytropolones Inhibit Initiation of Hepatitis B Virus Minus-Strand DNA Synthesis

**DOI:** 10.3390/molecules25194434

**Published:** 2020-09-27

**Authors:** Ellen Bak, Jennifer T. Miller, Andrea Noronha, John Tavis, Emilio Gallicchio, Ryan P. Murelli, Stuart F. J. Le Grice

**Affiliations:** 1 Basic Research Laboratory National Cancer Institute, Frederick, MD 21702, USA; ellen.bak@nih.gov (E.B.); millerj@mail.nih.gov (J.T.M.); andreanoronha1@gmail.com (A.N.); 2 Department of Molecular Microbiology and Immunology, St. Louis University, St. Louis, MO 63104, USA; john.tavis@health.slu.edu; 3 Department of Chemistry, Brooklyn College, The City University of New York, Brooklyn, NY 11210, USA; EGallicchio@brooklyn.cuny.edu (E.G.); RPMurelli@brooklyn.cuny.edu (R.P.M.); 4 PhD Program in Chemistry, The Graduate Center of The City University of New York, New York, NY 10016, USA; 5 PhD Program in Biochemistry, The Graduate Center of The City University of New York, New York, NY 10016, USA

**Keywords:** Hepatitis B virus, protein priming, epsilon RNA, minus strand DNA synthesis, 3,7-dihydroxytropolones

## Abstract

Initiation of protein-primed (-) strand DNA synthesis in hepatitis B virus (HBV) requires interaction of the viral reverse transcriptase with epsilon (ε), a *cis*-acting regulatory signal located at the 5’ terminus of pre-genomic RNA (pgRNA), and several host-encoded chaperone proteins. Binding of the viral polymerase (P protein) to ε is necessary for pgRNA encapsidation and synthesis of a short primer covalently attached to its terminal domain. Although we identified small molecules that recognize HBV ε RNA, these failed to inhibit protein-primed DNA synthesis. However, since initiation of HBV (-) strand DNA synthesis occurs within a complex of viral and host components (e.g., Hsp90, DDX3 and APOBEC3G), we considered an alternative therapeutic strategy of allosteric inhibition by disrupting the initiation complex or modifying its topology. To this end, we show here that 3,7-dihydroxytropolones (3,7-dHTs) can inhibit HBV protein-primed DNA synthesis. Since DNA polymerase activity of a ribonuclease (RNase H)-deficient HBV reverse transcriptase that otherwise retains DNA polymerase function is also abrogated, this eliminates direct involvement of RNase (ribonuclease) H activity of HBV reverse transcriptase and supports the notion that the HBV initiation complex might be therapeutically targeted. Modeling studies also provide a rationale for preferential activity of 3,7-dHTs over structurally related α-hydroxytropolones (α-HTs).

## 1. Introduction

Global estimates indicate that ~270 million individuals are chronically infected with hepatitis B virus (HBV) [1], experiencing liver diseases such as cirrhosis and hepatocellular carcinoma which, cumulatively, account for ~600,000 annual deaths [1,2]. HBV, a member of the *Hepadnaviridae* family, is the smallest animal-infecting DNA virus, with a ~3.2 Kb genome comprising seven proteins encoded by four overlapping genes [3,4]. HBV reverse transcriptase (P protein), the only enzyme of this genome, comprises a terminal protein (TP) linked to the reverse transcriptase (RT)/ribonucleaseH (RNase H) components by a spacer domain [5]. Following infection, partially double-stranded relaxed circular (rc) DNA is repaired in the nucleus, yielding covalently closed circular DNA (cccDNA), the transcriptional template for host RNA polymerase II. Viral transcripts are transported to the cytoplasm and translated into the core, pre-core protein, that is proteolytically processed and secreted as e-antigen, polymerase, envelope, and X proteins. Single-stranded pre-genomic RNA (pgRNA) is then packaged into the core during assembly of viral nucleocapsids and subsequently reverse-transcribed into minus-strand DNA [3,6]. Due to its genome size and encoding of only a single enzyme, developing effective strategies to treat HBV infections has been challenging [2]. Current options include immunomodulatory agents, such as interferon-α and pegylated interferon-α, or oral nucleoside/nucleotide analogues such as lamivudine, adefovir, telbivudine, entecavir, and tenofovir [7]. However, the potential side effects following decades of drug exposure suggests a need for novel strategies, and possibly their incorporation into combination therapies [8].

As with retroviral RTs, degradation of the RNA/DNA HBV replication intermediate is catalyzed by its RT-associated RNase H domain. Although no inhibitor of HIV-1 RT RNase H function has advanced to the clinic, intense efforts over almost 2 decades have made a plethora of compounds available for testing its HBV counterpart [9,10]. Following reports that the natural product α-hydroxytropolone (α-HT), β-thujaplicinol [11] inhibits HBV replication by sequestering the catalytic Mg^++^ in the RNase H active site [12], modified α-HTs [13], 2-hydroxyisoquinoline-1,3(2H,4H)-diones [14] and *N*-hydroxypyridinediones, have emerged as a new class of RNase H inhibitors [15]. The availability of an active form of the isolated HBV RNase H domain [16] should also promote development of high-throughput assays to accelerate drug-screening efforts.

Participation of several host factors is essential for early events of HBV minus-strand DNA synthesis, an example of which is the Hsp90 complex [17,18]. It is therefore not unreasonable to postulate that inducing dissociation of the multi-component initiation complex, or altering its topology, might be exploited to abrogate HBV infection therapeutically [17]. With this in mind, we have adopted the reconstituted in vitro HBV protein priming assay [19] to screen several classes of structurally diverse small molecules for their ability to inhibit priming of HBV DNA synthesis. Surprisingly, two classes of ligands that recognized the “priming loop” of HBV epsilon ε [6] failed to inhibit minus-strand DNA synthesis. The α-HT β-thujaplicinol, previously shown to inhibit HBV RNase H activity, had likewise little effect on initiation of DNA synthesis. In contrast, 3,7-dihydroxytropolones (3,7-dHTs), differing from α-HTs in that they that bear an extra, contiguous oxygen atom on their heptatriene ring, significantly reduced priming activity. Our findings that 3,7-dHTs inhibit protein priming by an RNase H-deficient HBV RT that otherwise retains DNA polymerase function, suggests they target the initiation complex in an RNase H-independent manner. Analyzing the composition of the priming complex also indicates that it remains intact in the presence of 3,7-dHT **196**. Our data therefore lends support to a recent proposal of Jones et al., who have demonstrated that the triphosphate form of the nucleoside analog Clevudine can inhibit priming of HBV minus-strand DNA synthesis via binding to and distorting the DNA polymerase active site of P protein [20].

## 2. Results

### 2.1. Establishing the HBV Minus-Strand DNA Priming System

**Figure 1:**
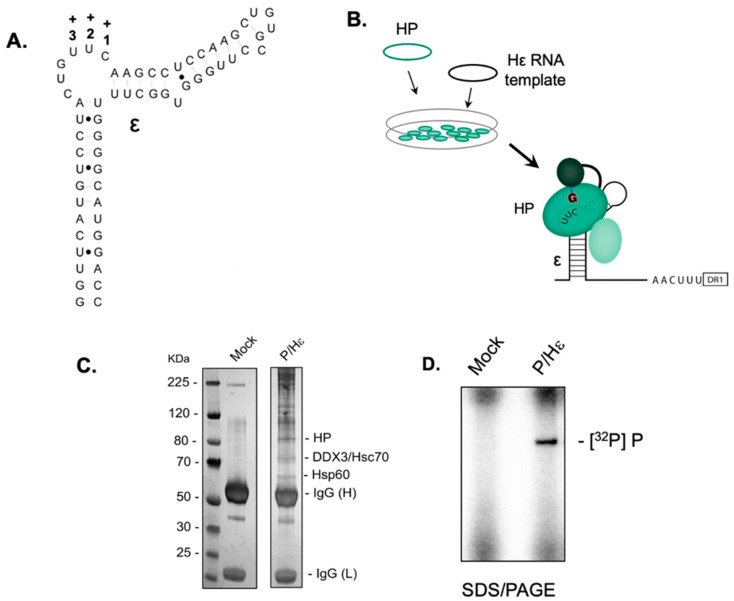
Establishing the in vitro HBV P protein priming system. (**A**) Structure of HBV ε RNA, outlining the priming loop and the site at which minus-strand DNA is initiated (**+1, +2, +3**). (**B**) Experimental HBV minus-strand priming strategy. HEK293T cells are transfected with plasmid pCMV-3FHP (protein components, green), or in combination with plasmid pCMV-Hε (RNA component, black), and a clarified homogenate is immobilized on anti-FLAG beads. (**C**) Identification of cellular factors of the initiation complex. The lane designated “Mock” represents an immobilized homogenate of non-transfected cells. Migration positions of cellular factors are taken from Jones et al. [19], HP, HBV polymerase. (**D**) P protein-primed, ε-dependent initiation of HBV minus-strand DNA synthesis, reflected by HBV polymerase (P) as the sole [^32^P]-labeled protein of the priming mixture.

ε-Dependent initiation of HBV minus-strand DNA synthesis requires, in addition to the viral polymerase, several eukaryotic chaperones [6,19]. Attempts to reconstitute the priming system from purified components have been unsuccessful, leading Jones et al. to develop a recombinant mammalian system using HEK293T cells transfected with plasmids expressing FLAG-tagged HBV polymerase and ε RNA (Figure 1A, B, respectively) [19]. Figure 1C analyzes bead-bound proteins in the absence and presence of the HBV P protein-expressing plasmid by sodium dodecyl sulfate/polyacrylamide gel electrophoresis (SDS/PAGE). While we observed low-level non-specific binding in the mock transfection, P protein and associated cellular chaperones could be visualized following transfection with the HBV Pol plasmid. Data of Figure 1D demonstrates that the reconstituted, immobilized priming complex supported ε-dependent protein priming.

### 2.2. Small Molecules that Recognize HBV Epsilon Do not Inhibit Priming

**Figure 2:**
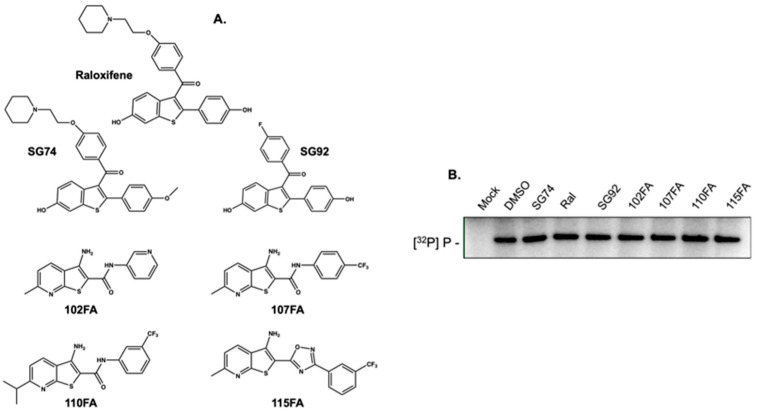
Structurally diverse ligands that identify the HBV ε priming loop fail to inhibit protein priming. (**A**) Structures of the SERM Raloxifene, derivatives SG74 and SG92 and HIV-1 TAR-binding analogs 102FA, 107FA, 110FA, and 115FA. (**B**) ε-dependent initiation of HBV minus-strand DNA synthesis in the presence of the analogs of (**A**). All analogs were tested at a final concentration of 100 μM.

As a feature of our ongoing studies to identify novel ligands that recognize *cis*-acting regulatory RNAs, we recently solved the structure of the 61-nt HBV ε RNA by NMR and employed a small-molecule microarray strategy to identify several ε-binding ligands (LeBlanc et al., manuscript submitted). These included the serum estrogen receptor modifier (SERM) Raloxifene, two related in-house synthesized analogs (SG74 and SG92), and human immunodeficiency virus transactivation response element (HIV-1 TAR)-binding ligands 102FA, 107FA, 110FA, and 115FA [21,22], which showed micromolar affinity for ε (Figure 2A). Since these ligands were selected on their interaction with the ε priming loop, our rationale was that accessing this structured RNA would hinder formation of the initiation complex. However, data of Figure 2B indicates that all ε-binding ligands failed to inhibit initiation of minus-strand DNA synthesis. Although not shown here, denaturing polyacrylamide gel electrophoresis indicated that ε RNA remained bound to immobilized HBV P protein in the presence of a ligand. While surprising, the outcome of Figure 2B likely reflects the low affinity of ε-binding ligands (20–50 μM) versus significantly greater affinity of HBV polymerase for ε which studies with nucleic acid polymerases such as HIV-1 RT predict would be in the low nanomolar range [23].

### 2.3. 3,7 Dihydroxytropolones Inhibit HBV (-) Strand DNA Synthesis

**Figure 3:**
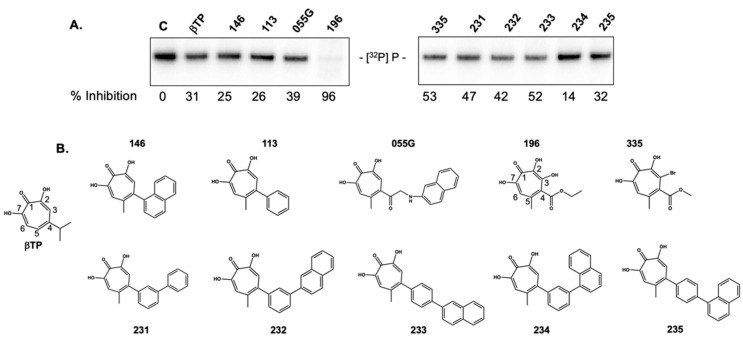
Sensitivity of ε-dependent initiation of HBV minus-strand DNA synthesis to α-HT and 3,7-dHT inhibitors previously shown to inhibit HBV and HIV RNase H activity. (**A**) Protein-primed, ε-dependent initiation of HBV minus-strand DNA synthesis. % inhibition at a ligand concentration of 100 μM is provided. (**B**) α-HT and 3,7-dHT structures. βTP, β-thujaplicinol. Ring numbering of βTP and the sole 3,7-dHT, **196**, has been provided for clarity.

Figiel et al. have demonstrated that, under conditions where the DNA polymerase active site of HIV-1 RT is covalently liked to its substrate, nucleic acid can be simultaneously accessed by the RNA polymerase and RNase H active centers, indicating an important degree of coordination between its synthetic and hydrolytic activities [24]. Using this precedent, we speculated that ligand binding at the HBV RNase H domain might allosterically modulate P protein DNA polymerase activity, since we and others have demonstrated that α-HTs inhibit HBV RNase H activity in vitro, and virus replication in culture [25,26,27]. In addition, Didierjean et al. have reported that DNA polymerase activity of HIV-1 RT can be inhibited by structurally related 3,7-dHTs [28]. Based on these studies, we elected to evaluate whether α-HTs and 3,7-dHTs inhibited initiation of HBV DNA synthesis, the results of which are presented in Figure 3.

**Figure 4:**
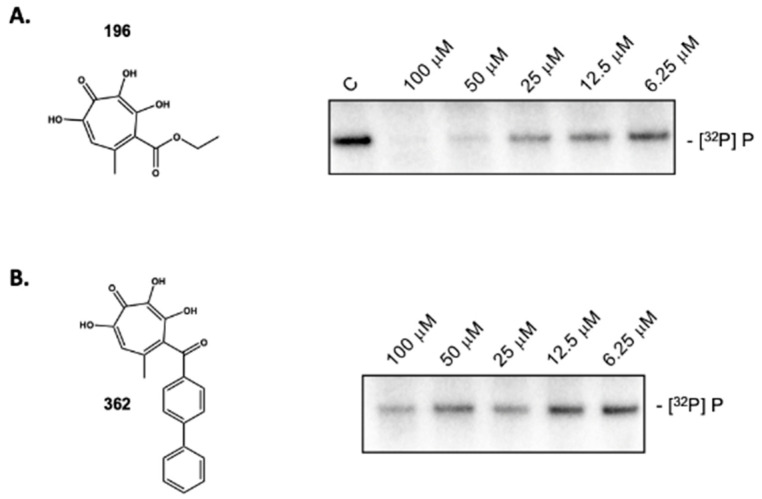
Inhibition of ε-dependent initiation of HBV minus-strand DNA synthesis by 3,7-dHTs. (**A**) **196** and (**B**) **362**. A dose-response analysis is presented for each compound.

**Figure 5:**
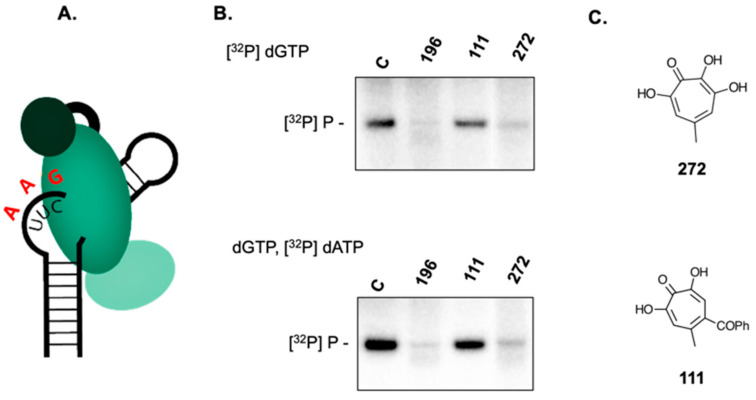
3,7-dHTs inhibit initiation of ε-dependent initiation of HBV minus-strand DNA synthesis and subsequent elongation. (**A**) Cartoon of the priming event. The trinucleotide sequence -C-U-U-(black) represents template nucleotides +1, +2 and +3, respectively. Inclusion of dGTP alone monitors initiation of DNA synthesis, while a mixture of dGTP and dATP (red) evaluates subsequent elongation steps. (**B**) dGTP (upper) and dGTP/dATP-primed reactions (lower) in the presence of 3,7-dHTs **196** and **272** and a control α-HT **111**. (**C**) structures of 3,7-dHT **272** and α-HT **111**. All analogs were tested at a final concentration of 100 μM.

Surprisingly, the α-HT β-thujaplicinol and several derivatives substituted at position 4 of the tropolone heptatriene ring had a minimal effect on priming of HBV DNA synthesis despite blocking production of the viral plus-polarity DNA strand due to RNase H inhibition, suggesting that ligand occupancy of the RNase H domain does not appear to translate to a significant conformational change at the DNA polymerase active site. In contrast, priming activity was reduced ~96% by the 3,7-dHT **196**. The requirement for four consecutive oxygen atoms was evident from the inactivity of compound **335**, which replaced the position 3-OH group with -Br. Figure 4 provides a dose-response curve for 3,7-dHT **196**, suggesting an IC_50_ in the 12.5–25 μM range. Although qualitative, 3,7-dHTs **362**, which has a bulkier diphenylketone, also demonstrated inhibitory activity at comparable concentrations. Coupled with the activity of the structurally simple **272** demonstrated in Figure 5B, the implication is that it is the 3,7-dHT core of four consecutive oxygen atoms is primarily responsible for their activity.

### 2.4. 3,7-dHTs Inhibit Covalent Attachment of dGTP to HBV P Protein

Early events of HBV (-) strand DNA synthesis might be considered a two-step process, whereby initiation via covalent linkage of dGTP to Tyr^63^ of P protein is followed by phosphodiester bond formation that attaches dATP and liberates pyrophosphate, after which RNA-dependent DNA synthesis ensues. Priming reactions performed in the presence of [^32^P]dATP would therefore not differentiate between the initiation and elongation steps. Defining the block to (-) strand DNA synthesis targeted by 3,7-dHTs therefore required comparing priming reactions in the presence of [^32^P]dGTP (minus DNA nt + 1) with those containing [^32^P]dATP (minus DNA nts + 2, +3, Figure 5A). The results of this strategy for compound **196** are presented in Figure 5B and compared with the simpler 3,7-dHT **272** and the αHT **111** (Figure 5C). As highlighted in Figure 5B, both 3,7-dHTs inhibit minus-strand DNA synthesis primed by [^32^P]dGTP alone and the dGTP/[^32^P]dATP mixture, while αHT **111** failed to inhibit DNA synthesis under either dNTP combination. The combined data of Figure 5 thus suggests that 3,7-dHTs antagonize covalent linkage of dGTP to HBV P protein. Mechanistically however, our data cannot distinguish between direct interaction with HBV P protein or antagonizing another component of the initiation complex that facilitates the priming reaction.

### 2.5. 3,7-dHTs Inhibit Priming by an RNase H-Deficient HBV Polymerase

**Figure 6:**
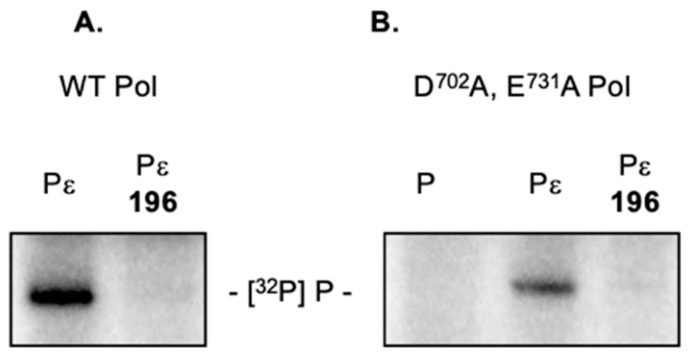
3,7-dHT **196** inhibits [^32^P]dGTP-primed, ε-dependent initiation of minus-strand DNA synthesis by HBV P protein mutant devoid of RNase H activity. (**A**) Wild type HBV P protein. (**B**) RNase H-deficient HBV P protein. Lanes Designated Pε represent the fully reconstituted priming reaction, while lanes designated P lack HBV ε RNA. 3,7-dHT **196** was used at a final concentration of 100 μM in both experiments.

Although the HBV P protein-encoded C-terminal RNaseH domain shares reduced homology with its counterpart enzymes from retroviruses and long-terminal repeat retrotransposons, a metal-binding motif common to the nucleotidyltansferase superfamily of nucleases [29] has been identified, i.e., -Asp^702^-Glu^731^-Asp^750^-Asp^790^-. Introducing point mutations at any of these positions destroys RNase H activity of the recombinant enzyme in vitro and virus replication in culture [30]. As a complementary approach to eliminate any indirect involvement of ligand binding to the HBV RNase H domain, priming reactions were reconstituted using an HBV P protein mutant carrying two RNase H-inactivating mutations, namely Asp^702^Ala and Glu^731^Ala [30]. Although we observed slightly lower priming activity with RNase H-deficient P protein, data of Figure 6B shows that this was also severely reduced in the presence of 3,7-dHT **196**. Since data from retroviral enzymes predicts that the dual Asp^702^Ala/Glu^731^Ala mutations likely lead to loss of divalent metal binding, the combined data of Figure 5 and Figure 6 rule out a direct contribution from the HBV RNase H domain.

### 2.6. Activity of Non-Troponoid Nucleotidyltransferase Inhibitors

**Figure 7:**
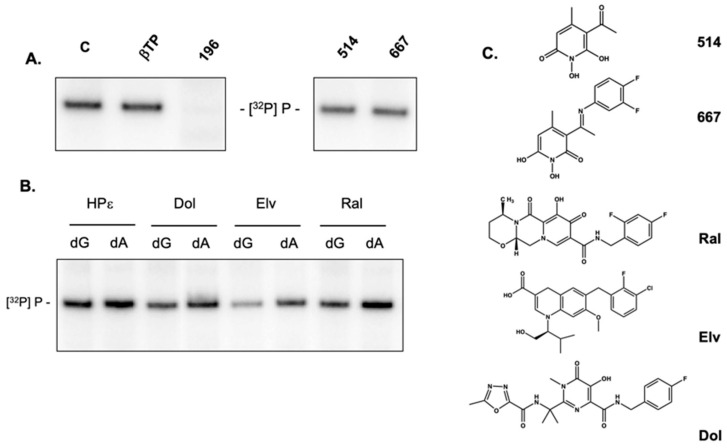
Inhibition of HBV ε-dependent initiation minus-strand DNA synthesis by non-tropanoid chemotypes that have been demonstrated to antagonize its RNase H activity. (**A**) Priming reactions performed in the presence of 3,7-dHT **196** and the *N*-hydroxypyridinediones **514** and **517**. (**B**) Priming reactions performed in the presence of diketo acid-based HIV integrase inhibitors Dolutegravir (Dol), Elvitegravir (Elv), and Raltegravir (Ral). (**C**) Structures of *N*-hydroxypyridinediones and diketo acids. All compounds were evaluated at a final concentration of 100 μM.

The N-naphthyridinone GSK364735, which shares a similar geometry and chelating function with α-HTs, has been reported as a potent inhibitor of HIV-1 integrase by binding competitively to the two-metal binding site of the integrase-HIV DNA complex [31,32]. Based on their mechanistic similarity as enzymes of the nucleotidyltransferase superfamily, Tavis et al. subsequently showed that both naphthyridinone- and *N*-hydroxypyridinedione-derived HIV-1 integrase inhibitors antagonized activity of recombinant HBV RNase H [30]. These observations prompted us to investigate whether representative *N*-hydroxypyridinediones inhibited priming of HBV DNA synthesis. As illustrated in Figure 7A, and similar to α-HTs, *N*-hydroxypyridinediones **514** and **667** were inactive, reinforcing the notion that the four consecutive oxygens of 3,7-dHTs were critical to stably sequester divalent metal at the DNA polymerase active site.

Tavis et al. also demonstrated efficacy of the diketo acid-derived integrase inhibitors Raltegravir and Elvitegravir as HBV RNase H inhibitors [30]. These and Dolutegravir were therefore examined in the HBV priming assay. Figure 7B examines [^32^P]dGTP-primed and [^32^P]dATP-primed reactions in the presence of Raltegravir, Elvitegravir, and Dolutegravir, comparing this with the 3,7-dHT **196**. Of these integrase inhibitors, Elvitegravir invoked a slight diminution of priming activity in the presence of either [^32^P]dGTP or [^32^P]dATP, while Raltegravir and Dolutegravir were essentially inactive.

### 2.7. 3,7-dHT 196 Does Not Induce Dissociation of the HBV Initiation Complex

The multi-protein nature of the HBV initiation complex [19] raised the possibility that inhibition of priming by 3,7-dHTs might reflect (a) disruption and release of one or more of the protein constituents, or (b) dissociation of the multi-protein complex from HBV ε RNA. To investigate this experimentally, we examined the nucleic acid and protein components of the immobilized initiation complex in the absence and presence of 3,7-dHT **196**. In Supplementary Figure S1A, ε RNA was visualized following denaturing PAGE by SYBR Gold staining, or indirectly by reverse transcription with a Cy5 end-labeled primer (Supplementary Figure S1B). In both instances, ε RNA was detected following incubation with 3,7-dHT **192**, confirming that it was neither degraded nor displaced from the initiation complex. Alternatively, following release of the bead-immobilized initiation complex, its constituents were analyzed by SDS/PAGE and silver staining. This analysis is illustrated in (Supplementary Figure S1C), where relevant proteins of the complex have been assigned according to Jones et al. [19]. While qualitative, silver staining shows minimal difference in eluates of extracts containing HBV polymerase alone, HBV polymerase complexed with ε or the HBV polymerase/ε complex incubated in the presence of 3,7-dHT **196**, suggesting that protein components of the priming complex are not perturbed in the presence of inhibitor.

### 2.8. Modeling Supports Additional Interactions of 3,7-dHTs at the HBV Polymerase Active Site

**Figure 8:**
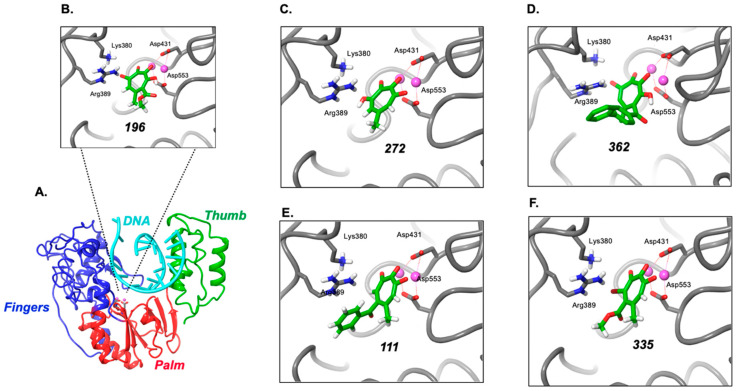
Modeling suggests a better “fit” of 3,7-dHTs that α-HTs at the DNA polymerase active site of HBV P protein. (**A**) Model for the fingers (blue)/palm (red)/thumb (green) domains of HBV polymerase containing double-stranded DNA (cyan). Divalent metals within the palm subdomain are indicated in magenta. (**B**–**D**) proposed binding poses of the 3,7-dHTs **196**, **272**, and **382**, respectively. Arg^389^, which is proposed to contact the fourth oxygen of the troponoid ring, is indicated, in (**E**,**F**) binding poses for the α-HTs **111**, and **335**, respectively.

Since a high-resolution crystal structure for the intact enzyme is unavailable, Das et al. created a three-dimensional model for the polymerase domain of HBV P protein (residues 325–699), based on homology modeling with the RTs of human immunodeficiency and murine leukemia virus [33]. Although sharing only 25% sequence identity, functionally important residues are conserved between the three enzymes. A representation of the HBV fingers/palm/thumb unit common to nucleic acid polymerases is outlined in Figure 8A. To better understand their specificity, 3,7-dHTs **196**, **272**, and **362** were docked to this homology model. In each case, a structure was revealed wherein three of the oxygens coordinate to the two metals in the active site, and the fourth oxygen engages in a hydrogen bond or salt bridge with Arg^389^ (Figure 8B–D, respectively). By removing one of the oxygens, the molecule revealed a primary binding pose that engaged Arg^389^, but only two oxygens could engage with the catalytic metals (α-HTs **111** and **335**, Figure 8E, F). A second pose positioned three oxygens to engage both metals, but an interaction with Arg^389^ was observed. While speculative, tridentate binding to the two catalytic metals in the DNA polymerase active site, combined with favorable interactions with Arg^389^, could facilitate stronger binding of 3,7-dHTs and inhibition of priming compared to α-HTs. Additional information is needed to validate this theory, and such studies are ongoing.

## 3. Discussion

Among the many steps in the HBV life cycle that might be considered therapeutically accessible, nucleoside and nucleotide analogs presently play a prominent role by antagonizing activity of the viral polymerase, evidenced by approval of the drugs lamivudine (3TC), adefovir dipivoxil (ADV), tenofovir (TDF) [34], entecavir (ETV), and telbivudine (LdT) [35]. Mechanistically, these chemotypes act as chain terminators through their inability to be extended following incorporation into nascent DNA. As a complement to chain termination, and with combination therapy in mind, inhibition of HBV P protein-associated RNase H is gaining increasing attention, evidenced by promising results with α-HTs and hydroxyimide chemotypes [9]. However, the multi-component nature of the HBV minus-DNA initiation complex, requiring a combination of viral and host proteins [17,18] and a unique conformation dissimilar to that catalyzing subsequent DNA strand elongation, raises alternatives to direct active site inhibition, such as (a) allosteric inhibition by sequestering the initiation complex to alter its overall topology or induce dissociation of a critical component [36], or (b) non-competitive inhibition, an example of which is foscarnet, which occupies the pyrophosphate-binding site on the viral enzyme [37]. Another encouraging example of noncompetitive inhibition has been proposed by Jones et al. [20], who demonstrated that Clevudine triphosphate (a derivative of thymidine triphosphate) inhibits HBV minus-strand DNA priming by distorting the active site of viral P protein in a manner incompatible with polymerization, which would be analogous to nonnucleoside RT inhibitors (NNRTI) of HIV RT [38]. With these issues in mind, we elected to establish the in vitro HBV priming system of Figure 1 to evaluate several chemotypes that have arisen from our HTS efforts to identify small-molecule protein and RNA-binding antagonists.

Based on the requirement for divalent metal (Mg^++^) at the DNA polymerase and RNase H active sites of HBV P protein, α–HTs, which we [11], and others [9,13,15,25,26,39] have studied extensively as viral RNase H inhibitors, were a logical starting choice. However, despite showing good activity against recombinant HBV RNase H, α–HTs were poorly active in inhibiting minus-strand priming. In contrast, we have demonstrated here that (a) structurally related 3,7-dHTs (compounds **196**, **272**, and **362**) inhibit priming and (b) the most promising, compound **196**, inhibits priming activity of an RNase H-deficient, polymerase-proficient HBV P protein. The requirement for an additional -OH group at position 3 of the tropolone ring is also suggested by lack of priming activity of compound **335**, which replaces this with -Br. A second class of non-tropolone HBV RNase H inhibitors, *N*-hydroxypyridinediones, also fails to inhibit priming, illustrating that the effect demonstrated here is specific to 3,7-dHTs. Since analysis of the RNA and protein components of the initiation complex suggest it remains intact (Supplementary Figure S1), we conclude that the 3,7-dHTs analyzed in this communication likely act through sequestration of divalent metal at the DNA polymerase active site. Indeed, this postulate is not without precedent, since Didierjean et al. have reported that although they antagonize RNase H activity of HIV-1 RT, 3,7-dHTs [28] are more specific for DNA polymerase function.

According to the 2-metal-ion catalysis mechanism proposed by Steitz and Steitz [40], the catalytic Mg^++^ ions at the RNase H active site of HIV-1 RT are separated by ~4Å, which Didierjean et al. have proposed is unfavorable for the interaction with tropolones [28]. However, in the complex of HIV-1 RT containing duplex DNA and the incoming dNTP [41], the two Mg^++^ ions in the DNA polymerase active site are separated by 3.57Å, which would be more in line with the 3.7Å separation distance of the two ions coordinated by 3,7-dHTs. Based on inhibition and modeling studies with inositol monophosphatase, Piettre et al. have proposed that three of the four contiguous oxygen atoms of 3,7-dHTs permit tridentate engagement with Mg^++^ ions, while the fourth oxygen atom is able to engage favorably with a main chain carbonyl group within the active site [42]. The fourth oxygen of the tropolone ring may similarly establish favorable contacts in the polymerase active site of HBV P protein, which modeling suggests could be Arg^389^. While detailed structural analysis will be necessary to validate these hypotheses, the finding of two chemotypes (Clevudine and 3,7-dHTs) that target the critical first step in HBV minus-strand DNA synthesis should spur new efforts to identify a novel class of therapeutic agents to target the HBV initiation complex. Since all studies reported here are based on an in vitro priming assay, future efforts should focus on determining how the activity of 3,7-dHTs we have identified translate into inhibition of HBV replication in infected cells.

## 4. Materials and Methods

### 4.1. α–HTs and 3,7-dHTs

Compounds **196**, **272**, and **362** were reported Hirsch et al. [39] (as compounds 6a, 6c, and 6e, respectively), **111**, **113**, **146**, and **335**, by Lomonosova et al. [26], compounds **231**, **232**, **233**, **234**, and **235** by Berkowitz et al. [43], and compound **055G** by D’Erasmo et al. [44] (as compound 4n). *N*-hydroxypyridinediones **514** and **667** have been reported by Tavis et al. [30].

### 4.2. Plasmids

Recombinant plasmids expressing 3× FLAG-tagged, full-length HBV DNA polymerase (pCMV-3FHP) and Hε RNA (pCMV-HE), derived from the 5′ end of HBV pgRNA, were a generous gift from Dr. Jianming Hu, Penn State University, College of Medicine, State College, PA, USA, and whose construction is described in Jones et al. [19]. Plasmid for expressing RNase H-deficient HBV polymerase (Asp^702^Ala/Glu^731^Ala) was prepared using a QuikChange Multi Site-Directed Mutagenesis kit (Agilent, Santa Clara, CA, USA) using the following primers:

Forward primer 5′-CCAAGTGTTTGCTGCCGCAACCCCCACTG-3′,

Reverse primer 5′-CGATCCATACTGCGGCACTCCTAGCCGCTTG-3′,

RNase H-inactivating mutations [30] were verified by DNA sequencing.

### 4.3. Protein, RNA Expression and Purification

HEK293T cells were transfected with pCMV-3FHP only (HBV polymerase, or P), or in combination with pCMV-Hε (RNA) using the Lipofectamine procedure (Invitrogen, Carlsbad, CA, USA). Two days post- transfection, cells were harvested as described in Reference [5]. P and P/ε complexes (PE) were purified with M2 anti-FLAG antibody (Millipore-Sigma, St. Louis, MO, USA) pre-bound to Protein A/G magnetic beads (ThermoFisher Scientific, Waltham, MA, USA), using 50 μL of FLAG beads per T75 flask (75 cm^2^) lysate (seeding with approximately 4.0 × 10^6^ cells 24 h prior to transfection). Beads were aliquoted into single-use portions and stored at −80 °C.

### 4.4. In Vitro Protein Priming

5 μL of P or Pε M2 beads/reaction were resuspended in priming buffer (20 mM Tris-HCl pH 7.0, 15 mM NaCl, 10 mM KCl, 4 mM MgCl_2_) with 1× (ethylenediaminetetraacetate) EDTA-free protease inhibitor cocktail (Millipore-Sigma), 2 mM dithiothreitol DTT), 1 mM phenylmethylsulfonyl fluoride (PMSF), and 1 U RNasin RNase inhibitor/μL buffer. Following 10-min pre-incubation with analogs and shaking at 25 °C, 0.5 μL of [α-^32^P]dGTP (10 mCi/mL (3000 Ci/mmol); PerkinElmer, Waltham, MA, USA) was added for a total volume of 20 μL/reaction, and mixtures were incubated at 25 °C for 1 h with shaking. Beads were washed twice in 300 mM NaCl, 0.05% Tween in 0.05 M Tris/HCl, Ph 7.6, 0.15 M NaCl (TBST), then incubated at 95 °C in 2× SDS sample buffer (Invitrogen) for 2 min. The supernatant was separated from beads using a magnetic rack and fractionated at room temperature through a 4–12% Bis-Tris polyacrylamide gel (200 V) in 1× MES running buffer (Invitrogen). To detect nucleotide incorporation at elongation and strand transfer, 0.5 μL of either [α-^32^P]dATP (10 mCi/mL (3000 Ci/mmol); PerkinElmer) was mixed with 6.5 nM unlabeled dGTP, or [α-^32^P] thymidine triphosphate (TTP) (10 mCi/mL (3000 Ci/mmol); PerkinElmer) was mixed with 6.5 nM unlabeled dGTP and 30 nM unlabeled dATP. Phosphorimaging was used to detect [^32^P]-labeled HP as the product of in vitro protein priming. Scanning was performed with a Typhoon FLA 9500 (GE Healthcare, Chicago, IL, USA) and quantitation used ImageQuant TL software (GE Healthcare).

### 4.5. In Vitro Binding of ε RNA to HBV Polymerase

M2 beads containing immobilized HBV P protein were resuspended in binding buffer (50 mM Tris/HCl, pH 7.5, 150 mM NaCl, 1 mM EDTA, 0.05% NP-40) with 1× complete with EDTA protease inhibitor cocktail (Millipore-Sigma), 1 mM PMSF, 2 mM dithiothreitol (DTT), and 1 U RNasin RNase inhibitor (Promega, Madison, WI, USA)/μL, and incubated with ligands for 10 min at 25 °C with shaking, followed by incubation with 1 M Cy5-labeled HBV ε RNA (137 nt) for 90 min at 25 °C in the dark with shaking. Beads were washed 4 times in TBST, then resuspended in 10 μL of 2× loading buffer (Invitrogen) and incubated at 90 °C for 3 min. Supernatant from the beads was fractionated through a 4–12% Bis-Tris gel in 1× MES running buffer as described above. Cy5-labeled ε RNA was detected and quantified as described above.

### 4.6. HBV ε RNA Detection

M2 beads containing HBV P protein bound to ε RNA were resuspended in priming buffer (5 μL of beads/reaction) and incubated with either DMSO (control) or 3,7-dHT **196** for 10 min at 25 °C, with shaking. Samples were next resuspended in 50 mM Tris/HCl, pH 8.0, 75 mM KCl and annealed at 85 °C with 2.5 pmole of Cy5-labeled HPε primer (5′ Cy5/CGAGAGTAACTCCACAGTAGCTCC 3′). After separating reaction supernatants from magnetic beads, a master mix was added to the supernatant for a final concentration of 0.5 mM deoxynucleoside triphosphates dNTPs), 3 mM MgCl_2_, 4 mM DTT. Urea gel loading buffer (1× Tris/Borate/EDTA containing 7 M Urea) was added to the T_0_ reactions prior to adding 50 U/μL SuperScript Reverse Transcriptase (ThermoFisher Scientific). For other time points, after addition of RT, samples were incubated at 45 °C for 10 min, denatured in 7 M urea loading buffer at 95 °C for 3 min, then placed on ice. Samples were resolved on a 6% polyacrylamide (19:1 acryl:bis)/7 M urea gel in 1× TBE. Cy5-labeled, reverse transcribed primer was detected as described above.

### 4.7. Modeling Ligand Binding within the HBV Polymerase Active Site

Molecular docking of compounds **111**, **196**, **272**, **335**, and **362** utilized the homology model structure of HBV bound to deoxycytidine triphosphate (dCTP) and a double-stranded DNA template primer proposed by Das et al. [33] as the receptor. The receptor was prepared using the Protein Preparation Wizard of the Maestro molecular modeling environment version 2019-4 (Schrödinger, Inc., New York, NY, USA). Residue G22 of chain F of the primer DNA strand was deleted to provide space for the incoming ligand and to mimic inhibition of elongation. The compounds were prepared using the LigPrep facility at neutral pH and default settings. The docking grid and subsequent ligand docking calculation were performed using the Glide program using Standard Precision and default settings, placing the center of the docking box at the location of the reference-bound dCTP. The binding pose with the lowest energy was retained for analysis.

## 5. Conclusions

Small molecules that recognize the “priming loop” of HBV ε RNA are unlikely to inhibit minus-strand DNA synthesis by inducing dissociation of the high-affinity, multi-protein initiation complex. In contrast, here, we showed that 3,7-dHTs, in contrast to the structurally related α–HTs, inhibit HBV P protein-primed initiation of minus-strand DNA, and provide a rationale for this by modeling the former into the P protein DNA polymerase active site. Combined with ongoing studies, this observation opens the notion of developing ligands that independently target the DNA polymerase and RNase H active sites of HBV P protein.

## Supplementary Materials

Figure S1: 3,7-dHT **196** does not comprise the integrity of the HBV priming complex.
