# Supplementary material for: 3,7-Dihydroxytropolones Inhibit Initiation of Hepatitis B Virus Minus-Strand DNA Synthesis
Source: Molecules. 2020 Sep 27;25(19):4434. doi: 10.3390/molecules25194434 (PMC7583054; doi:10.3390/molecules25194434)
Supplement: Supplementary file 1 [file molecules-25-04434-s001.pdf]

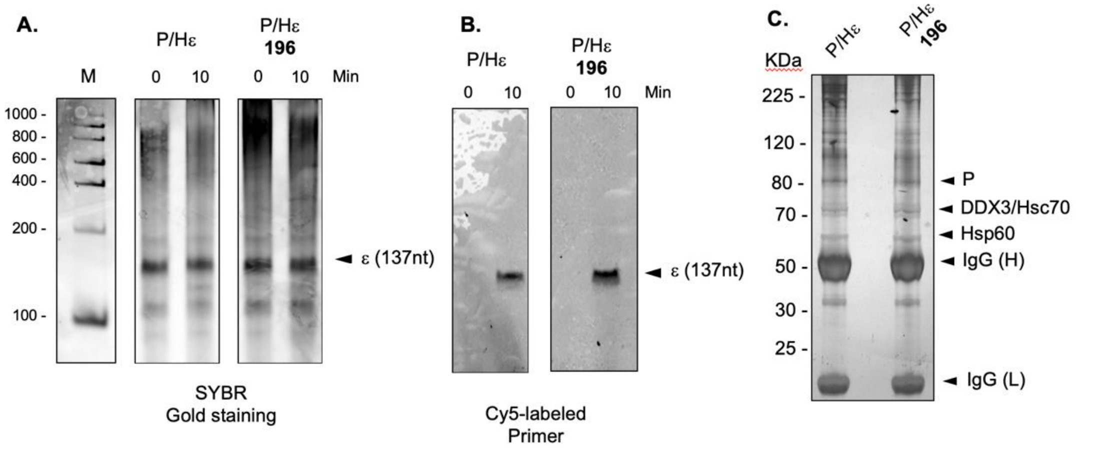

Supplementary Figure S1. 3,7-dHT **196** does not comprise the integrity of the HBV priming complex. **A, B,** Priming complexes were analyzed for HBV e RNA in the absence of, and following incubation with, 3,7-dHT **196** by either SYBR Gold staining (**A**) or reverse transcription with a Cy-5-labeled primer (**B**). **C**, Analysis of the protein components of the priming mixture in the absence of, and following incubation with, 3,7-dHT **196** by SDS/PAGE. In (**C**), The lane designated "P/He" is identical to that of Figure 1 **C**.
